# Characteristics and outcome profile of hospitalized African patients with COVID-19: The Ethiopian context

**DOI:** 10.1371/journal.pone.0259454

**Published:** 2021-11-09

**Authors:** Tigist W. Leulseged, Ishmael S. Hassen, Endalkachew H. Maru, Wuletaw C. Zewsde, Negat W. Chamiso, Abdi B. Bayisa, Daniel S. Abebe, Birhanu T. Ayele, Kalkidan T. Yegle, Mesay G. Edo, Eyosyas K. Gurara, Dereje D. Damete, Yared A. Tolera

**Affiliations:** Millennium COVID-19 Care Center, St. Paul’s Hospital Millennium Medical College, Addis Ababa, Ethiopia; Health Directorate, LUXEMBOURG

## Abstract

**Background:**

The COVID-19 pandemic seems to have a different picture in Africa; the first case was identified in the continent after it had already caused a significant loss to the rest of the world and the reported number of cases and mortality rate has been low. Understanding the characteristics and outcome of the pandemic in the African setup is therefore crucial.

**Aim:**

To assess the characteristics and outcome of Patients with COVID-19 and to identify determinants of the disease outcome among patients admitted to Millennium COVID-19 Care Center in Ethiopia.

**Methods:**

A prospective cohort study was conducted among 1345 consecutively admitted RT-PCR confirmed Patients with COVID-19 from July to September, 2020. Frequency tables, KM plots, median survival times and Log-rank test were used to describe the data and compare survival distribution between groups. Cox proportional hazard survival model was used to identify determinants of time to clinical recovery and the independent variables, where adjusted hazard ratio, P-value and 95% CI for adjusted hazard ratio were used for testing significance and interpretation of results. Binary logistic regression model was used to assess the presence of a statistically significant association between disease outcome and the independent variables, where adjusted odds ratio, P-value and 95% CI for adjusted odds ratio were used for testing significance and interpretation of results.

**Results:**

Among the study population, 71 (5.3%) died, 72 (5.4%) were transferred and the rest 1202 (89.4%) were clinically improved. The median time to clinical recovery was 14 days. On the multivariable Cox proportional hazard model; temperature (AHR = 1.135, 95% CI = 1.011, 1.274, p-value = 0.032), COVID-19 severity (AHR = 0.660, 95% CI = 0.501, 0.869, p-value = 0.003), and cough (AHR = 0.705, 95% CI = 0.519, 0.959, p-value = 0.026) were found to be significant determinants of time to clinical recovery. On the binary logistic regression, the following factors were found to be significantly associated with disease outcome; SPO2 (AOR = 0.302, 95% CI = 0.193, 0.474, p-value = 0.0001), shortness of breath (AOR = 0.354, 95% CI = 0.213, 0.590, p-value = 0.0001) and diabetes mellitus (AOR = 0.549, 95% CI = 0.337, 0.894, p-value = 0.016).

**Conclusions:**

The average duration of time to clinical recovery was 14 days and 89.4% of the patients achieved clinical recovery. The mortality rate of the studied population is lower than reports from other countries including those in Africa. Having severe COVID-19 disease severity and presenting with cough were found to be associated with delayed clinical recovery of the disease. On the other hand, being hyperthermic is associated with shorter disease duration (faster time to clinical recovery). In addition, lower oxygen saturation, subjective complaint of shortness of breath and being diabetic were associated with unfavorable disease outcome. Therefore, patients with these factors should be followed cautiously for a better outcome.

## Introduction

Over the past ten months the COVID-19 pandemic has caused a significant loss, both to the human life and the economy, all over the world. The Africa continent, which is already burdened with communicable diseases like HIV and tuberculosis with limited health care infrastructure and underdeveloped healthcare sector, seemed to be less affected by the pandemic. According to the World Health Organization weekly epidemiological report, as of April 13, 2021 globally there were 135,057,587confirmed cases. Africa constituted only 2% (3,171,006) of the global case. So far, a total of 79,545 deaths were reported in the continent and this constitutes only 3% of the global cumulative death. In Ethiopia, on the same day, there were a total of 227,255 confirmed cases and 3146 deaths [1].

Numerous studies are conducted globally to understand the pandemic better, but still studies are scarce in Africa. A study conducted in northern Ethiopia among hospitalized cases shows that in-hospital mortality was 0.8% and determined by older age, malignancy and surgery/trauma [2]. Studies conducted in Democratic Republic of Congo and Nigeria reported mortality rate that ranges from 4.3% up to 13.2% and age, body mass index, farming occupation, history of chronic kidney disease and symptoms of cough, shortness of breath and vomiting were implicated to be important predictors [3–5].

Different clinical presentation, disease course and outcome have been reported that differs from study to study and place to place implying the need for further study to understand the disease better. Regarding the clinical presentation, symptomatic and asymptomatic presentations have been reported during the entire disease course. In addition, the type of symptoms could vary manifesting as different body system symptoms with some presenting with what is called atypical presentations for a virus that attacks the respiratory system. The commonly reported symptoms are respiratory and constitutional symptoms [6–12].

The disease outcome is also reported to vary between a complete recovery without any complication, development of one or more systemic complication or death [13–15]. These disease outcomes seemed to be determined by underlying patient characteristics, history of pre-existing medical conditions, the COVID-19 disease severity, disease progression and the development of complications [14, 16–23].

Due to disparity in reported results from studies conducted in different countries, understanding the disease characteristics and its outcome in the local context is crucial for targeted intervention.

Therefore, in this study we aimed to assess the characteristics and outcome of 1345 RT-PCR confirmed Patients with COVID-19 and to identify the determinants of the disease outcome among patients admitted to Millennium COVID-19 Care Center in Ethiopia.

## Materials and methods

### Study setting, design and population

An institution based prospective cohort study was conducted at Millennium COVID-19 Care Center (MCCC), a 1000 bed makeshift hospital in Addis Ababa, Ethiopia dedicated for isolating and treating COVID-19 cases. The center is remodeled from the previous Millennium hall/ Addis park which was a multipurpose recreational, meeting and exhibition center. It is the first makeshift center and also the largest center in Ethiopia with majority of the cases in the country admitted to the center especially during the first few months of the pandemic before the other COVID-19 wings in permanent hospitals and regional centers started giving service. Even after that, it remained to be a center with the largest admission. At the beginning, since there were few cases, the MCCC and also other centers in the country were used as both a quarantine and treatment center in order to halt the transmission of the disease. Therefore, for the first few weeks after the cener started to give service on June 2, 2020, anyone who tested positive for SARS-Cov-2 used to be admitted to the center despite their age, disease severity, presence of symptoms and co-morbidity status.

The study was conducted from mid July, when the center started functioning with full capacity including intensive care admission and the admission was restricted to only symptomatic cases, up to the end of September, 2020.

The source population was all cases of COVID-19 admitted at MCCC with a confirmed diagnosis of COVID-19 using RT-PCR, as reported by a laboratory given mandate to test such patients by the Ministry of Health and who were on follow up from July to September, 2020 [24].

### Sample size determination and sampling technique

All consecutively admitted Patients with COVID-19 during the three months follow up period were included in the study.

During this interval a total of 1741 Patients with COVID-19 were admitted to the Center.

### Eligibility criteria

All Patients with COVID-19 who were on treatment and follow up at the center from July to September, 2020 and with complete follow up data were included.

### Operational definition

**COVID-19 disease** [25]**:**

- **Mild Disease:** characterized by fever, malaise, cough, upper respiratory symptoms, and/or less common features of COVID-19 (headache, loss of taste or smell etc…)
- **Moderate Disease:** Patients with lower respiratory symptom/s. They may have infiltrates on chest X-ray. These patients are able to maintain oxygenation on room air.
- **Severe COVID-19 disease:** Includes patients who have developed complications. The following features can define severe illness.
- ◦ Hypoxia: SPO2 ≤ 93% on atmospheric air or PaO2:FiO2 < 300mmHg (SF ratio < 315)
- ◦ Tachypnea: in respiratory distress or RR>30 breaths/minutes
- ◦ More than 50% involvement seen on chest imaging

**Clinical recovery:** implies resolution of symptoms and/ or signs of patients as evidenced by clinical, laboratory and radiologic assessments irrespective of the biochemical recovery.

**Event:** Clinical recovery from COVID-19.

**Censoring**: Includes patients who were lost to follow-up, transferred out, died or completed the follow-up period before clinical recovery.

**Time to event or censoring**: time between admission to the center up to clinical recovery or censoring (in days).

### Data collection procedures and quality assurance

Data was collected from patients and their medical charts using a pretested interviewer administered questionnaire. To improve data quality, training on the basics of the questionnaire and data collection tool was given for fifteen data collectors (General practitioners) and four supervisors (General practitioner and public health specialist) for two days. In addition, double data entry, and data cleaning through checking for inconsistencies, numerical errors and missing parameters was done. Where discrepancies are observed, data entered was verified with the primary data source. Where possible, data was validated by comparing a certain percentage of data in our database with that of another database. Data consistency and completeness was checked before an attempt was made to enter the code and analyze the data. Once data cleaning was complete, the data was exported to SPSS version 23 for analysis.

The recommended infection prevention and control practice was implemented during the data collection.

### Statistical analysis

The collected data was coded and entered into Epi-Info version 7.2.1.0, cleaned and stored and exported into SPSS version 23 for analysis. Data for continues variables was described and summarized using mean ± SD and for categorical variables data was expressed using proportions with frequency tables, Kaplan Meier (KM) plots and median survival times. Survival experience of different groups was compared using KM survival curves. Log-rank test was used to assess significant difference among survival distributions of groups for equality.

To assess the presence of a statistically significant association between the independent variables and the primary outcome (time to clinical recovery), multivariable Cox proportional hazard survival model was used. Univariate analysis at 25% level of significance was performed to calculate an unadjusted hazard ratio (HR) and to screen out potentially independent variables. In the final model; Adjusted HR, P-value and 95% CI for HR were used to test significance and interpretation of results. Variables with p-value ≤ 0.05 were considered to have a statistically significant association with time to clinical recovery in days. The basic assumptions of Cox Proportional Hazard model was tested using log minus log function, where parallel lines indicate proportionality, and the data fitted well.

Similarly, to assess the association between the relevant independent variables and the secondary outcome (disease outcome), multiple Binary logistic regression model was used. Univariate analysis at 25% level of significance was done to screen out independent variables to be used in the multiple Binary Logistic regression model. The adequacy of the final model was assessed using the Hosmer and Lemeshow goodness of fit test and the final model fitted the data well (p-value = 0.167). For the Binary Logistic regression, 95% confidence interval for AOR was calculated and variables with p-value ≤ 0.05 were considered to have a statistically significant association with disease outcome.

### Ethical considerations

The study was conducted after obtaining ethical clearance from St. Paul’s Hospital Millennium Medical College Institutional Review Board (Ref No. pm23/23).

Written informed consent was obtained from the participants. The study had no risk/negative consequence on those who participated in the study. Medical record numbers were used for data collection and personal identifiers were not used in the research report. Access to the collected information was limited to the principal investigator and confidentiality was maintained throughout the project.

## Results

### Disease outcome, censoring status and median time to clinical recovery

**Fig 1:**
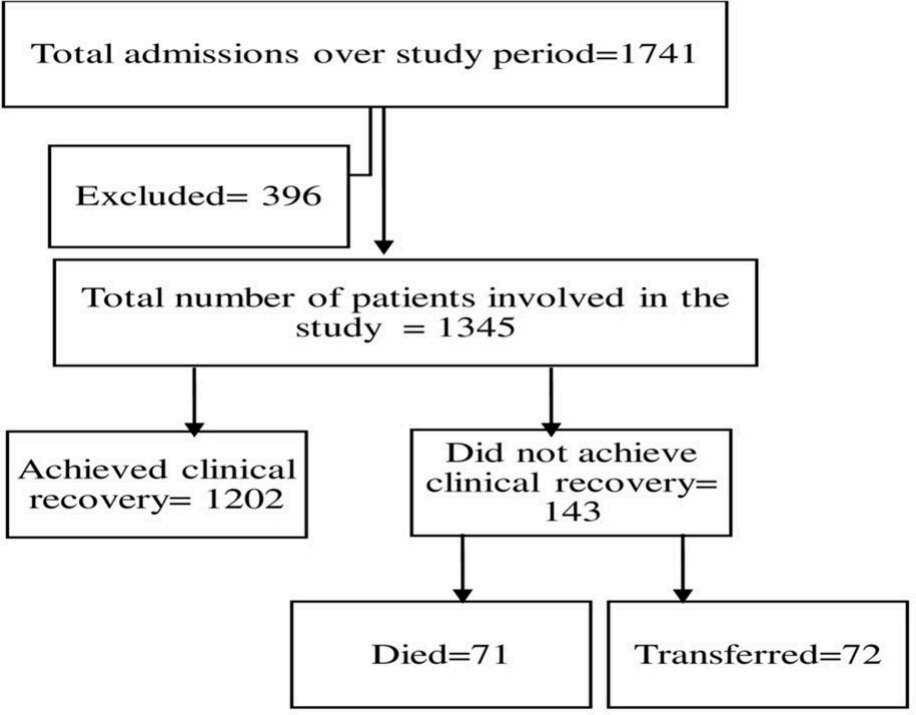
Flow chart showing the disposition of study participants in the final analysis.

Among the 1741 patients, 1345 (77.3%) patients with complete medical records were included in the study. The rest 396 charts were cases of mild and moderate COVID-19 with no records of more than half of study variables included in the analysis and therefore were excluded from the study. Seventy-one (5.3%, 95% CI = 4.1%-6.5%)) died, 72 (5.4%) were transferred and the rest 1202 (89.4%) were discharged improved. **(Fig 1)**

More than half (59.7%) of the transferred cases were female. Majority (63.9%) had mild disease, 14 (19.4%) had moderate disease and only 12 (16.7%) had severe disease. The 12 severe cases were transferred to another institution for further specialty care including dialysis service which was not available at the center during the study period. The rest were mainly transferred to other institutions due to patient and family request for convenience of visit.

Among the 1345 patients, 1202 (89.4%) achieved clinical recovery and 143 (10.6%) were censored. The overall median time to clinical recovery was 14 days.

### Socio-demographic, clinical and laboratory characteristics and censoring status of study participants

The mean age of the participants was 41.0 ± 18.2 years and 778 (58.6%) were males. The majority of the participants (92.9%) were from Addis Ababa, the capital city of Ethiopia. Only 26 (1.9%) of the participants were health care professionals. From the 557 females, 13 (2.3%) were pregnant.

Around one-third (34.2%) of the patients had a history of one or more pre-existing co-morbid illness. The major co-morbid illness among the participants was hypertension (19.9%), diabetes (13.7%), cardiac disease (4.2%) and asthma (4.1%). Chronic kidney disease, chronic liver disease, neurologic disorder and chronic pulmonary diseases constitute only 2.6% altogether.

Based on the vital sign recorded on triage, majority had SBP of < 140 (54.7%), DBP of < 90 (72.4%), temperature of > 37.5°C (52.3%) and oxygen saturation of 93% and above (80.4%).

In all of the variable categories, the number of those who achieved clinical recovery was greater than the censored observations. The proportion of censored observations was relatively larger as age increases, for males, for patients with one or more pre-existing comorbid illness and Khat chewers. On the other, those with a history of drug use had a relatively less censored observation than those with no drug use history.

A statistically significant difference in the time to clinical recovery was observed among patients based on age group, hypertension, cardiac disease, diabetes mellitus and oxygen saturation. Accordingly, the median duration of time to clinical recovery was significantly longer for patients 50 years and older (15 days) compared to those patients younger than 50 years of age (14 days). Having a pre-existing hypertension, cardiac disease (and diabetes mellitus (15 days Vs 14 days) was associated with a delayed clinical recovery compared to patients with no such comorbid illnesses. In addition, delayed clinical recovery was observed among patients whose oxygen saturation was lower than 93% at presentation (16 days Vs 14 days).

More than half of the patients (52.3%) were triaged to have mild COVID-19 severity disease and the rest were moderate (24.7%) to severe (23.0%).

According to the log-rank test result, a significantly longer time to clinical recovery was needed among patients with a complaint of fever (15 days Vs 14 days), chest pain (15 days Vs 14 days), nausea/vomiting (16 days Vs 14 days), fatigue (15 days Vs 14 days), shortness of breath (15 days Vs 14 days) and headache (15 days Vs 14 days). In addition, time to clinical recovery seemed to differ based on disease severity, with sever disease associated with a prolonged time compared to those patients with mild and moderate disease (16 days Vs 14 days). (**Table 1**)

**Table 1:** Socio-demographic, clinical and laboratory characteristics and censoring status of study participants (n = 1345).

Variable	Recovery		Total (%)	Time to recovery [median (IQR) days]	P-value
	Recovered (%)	Not Recovered (%)			
**Age**					
< 30	444 (94.8)	39 (8.1)	483 (35.9)	14.0	0.0001*
30–49	421 (93.2)	36 (7.9)	457 (34.0)	14.0	
≥ 50	337 (83.9)	68 (16.8)	405 (30.1)	15.0	
**Sex**					
Female	491 (90.3)	66 (11.8)	557 (41.4)	14.0	0.961
Male	711 (91.5)	77 (9.8)	778 (58.6)	14.0	
**SBP**					
< 140	681 (91.8)	79 (10.4)	706 (54.7)	14.0	0.733
≥ 140	520 (89.9)	64 (11.0)	584 (45.3)	14.0	
**DBP**					
< 90	876 (91.9)	98 (10.1)	974 (72.4)	14.0	0.195
≥ 90	326 (88.7)	45 (12.1)	371 (27.6)	14.0	
**Temperature**					
≤ 37.5°C	571 (90.8)	71 (11.1)	642 (47.7)	15.0	0.095
> 37.5°C	631 (91.2)	72 (10.2)	703 (52.3)	14.0	
**SPO2**					
≥ 93	1008 (95.0)	73 (6.8)	1081 (80.4)	14.0	0.0001*
< 93	194 (74.6)	70 (26.5)	264 (19.6)	16.0	
**Preexisting Co-morbid illness**					
No	813 (93.9)	72 (8.1)	885 (65.8)	14.0	0.053
Yes	389 (85.4)	71 (15.4)	460 (34.2)	14.0	
**Hypertension**					
No	973 (90.3)	105 (9.7)	1078 (80.1)	14.0	0.036*
Yes	229 (85.8)	38 (14.2)	267 (19.9)	15.0	
**Cardiac disease**					
No	1161 (91.8)	127 (9.9)	1288 (95.8)	14.0	0.019*
Yes	41 (71.9)	16 (28.1)	57 (4.2)	15.0	
**Diabetes Mellitus**					
No	1054 (92.5)	107 (9.2)	1161 (86.3)	14.0	0.005*
Yes	148 (81.5)	36 (19.6)	184 (13.7)	15.0	
**Asthma**					
No	1152 (91.0)	138 (10.7)	1290 (95.9)	14.0	0.901
Yes	50 (90.9)	5 (9.1)	55 (4.1)	14.0	
**Fever**					
No	992 (91.7)	110 (10.0)	1102 (81.9)	14.0	0.0001*
Yes	210 (87.7)	33 (13.6)	243 (18.1)	15.0	
**Cough**					
No	707 (95.5)	52 (6.9)	759 (56.4)	14.0	0.0001*
Yes	495 (85.2)	91 (15.5)	586 (43.6)	15.0	
**Nausea/ vomiting**					
No	1166 (91.1)	138 (10.6)	1304 (97.4)	14.0	0.026*
Yes	36 (97.8)	5 (12.2)	41 (3.0)	16.0	
**Sore throat**					
No	1065 (90.8)	131 (11.0)	1196 (88.9)	14.0	0.340
Yes	137 (92.6)	12 (8.1)	149 (11.1)	14.0	
**Runny nose**					
No	1125 (90.8)	138 (10.9)	1263 (93.9)	14.0	0.226
Yes	77 (93.9)	5 (6.1)	82 (6.1)	14.0	
**Chest pain**					
No	1058 (91.7)	119 (10.1)	1177 (87.5)	14.0	0.0001*
Yes	144 (86.3)	24 (14.3)	168 (12.5)	15.0	
**Myalgia**					
No	1070 (91.1)	128 (10.7)	1198 (89.1)	14.0	0.302
Yes	132 (91.5)	15 (10.2)	147 (10.9)	15.0	
**Arthralgia**					
No	1074 (91.3)	125 (10.4)	1199 (89.1)	14.0	0.160
Yes	128 (88.4)	18 (18.0)	146 (10.9)	15.0	
**Fatigue**					
No	984 (93.0)	97 (9.0)	1081 (80.4)	14.0	0.0001*
Yes	218 (83.0)	46 (17.4)	264 (19.6)	15.0	
**SOB**					
No	986 (94.9)	74 (7.0)	1060 (78.8)	14.0	**0.0001**
Yes	216 (76.5)	69 (24.2)	285 (21.2)	15.0	
**Headache**					
No	1041 (91.9)	115 (9.9)	1156 (85.9)	14.0	0.012*
Yes	161 (84.7)	28 (14.8)	189 (14.1)	15.0	
**COVID-19 Severity**					
Mild	656 (96.2)	47 (6.7)	703 (52.3)	14.0	0.0001*
Moderate	316 (95.5)	16 (4.8)	332 (24.7)	14.0	
Severe	230 (74.5)	80 (25.8)	310 (23.0)	16.0	

**Fig 2:**
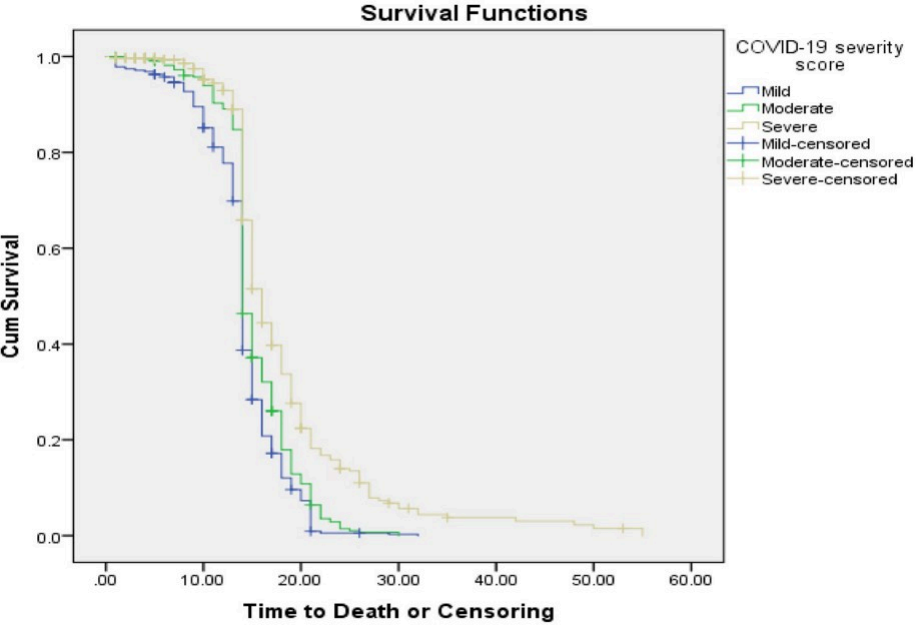
Kaplan-Meier survival analysis of recovery time stratified by COVID-19 clinical category/severity.

The Kaplan Meir (KM) survival function graphs also showed that those with mild disease have a favorable time to clinical recovery compared to those with more severe disease category. (**Fig 2**).

### Factors associated with time to clinical recovery

Crude analysis of each independent variable with the time to clinical recovery was run at 25% level of significance. From univariate analysis; age group, SPO2, temperature, COVID-19 severity, cough, chest pain, arthralgia, fatigue, headache, shortness of breath, hypertension and diabetes were significantly associated with time to clinical recovery among Patients with COVID-19.

However; only temperature, COVID-19 severity and cough were found to be significantly associated with time to clinical recovery in the multivariable Cox proportional hazard model at 5% level of significance. The proportional hazards assumption of the model was tested using the Log-minus-Log function on SPSS version 23 software. The plots show a reasonable fit to the assumption with Parallel lines between groups indicate proportionality [26].

Accordingly, after adjusting for other covariates, the rate of achieving clinical recovery among patients with temperature of 37.5°C and above was 1.135 times than patients with a temperature range of37.5°C and lower (AHR = 1.135, 95% CI = 1.011, 1.274, p-value = 0.032). This shows that hyperthermic patients improve from COVID-19 in a significantly shorter duration.

COVID-19 disease severity at admission was also found to be a significant determinant of time to clinical recovery. The rate of achieving clinical recovery among patients with severe COVID-19 disease at presentation was 34% lower than patients who presented with mild disease (AHR = 0.660, 95% CI = 0.501, 0.869, p-value = 0.003). On the other hand, there was no significant difference between moderate and mild cases in the time to clinical recovery. That means, having severe disease is associated with delayed clinical recovery.

Presenting with a complaint of cough at admission was associated with a 24.1% lower rate of achieving clinical recovery compared to those patients with no such complaint on admission (AHR = 0.705, 95% CI = 0.519, 0.959, p-value = 0.026) showing that having cough resulted in delayed clinical recovery (**Table 2**)

**Table 2:** Result of multivariable Cox proportional hazard model among patients with COVID-19 (n = 1345).

Variable	CHR (95% CI)	AHR (95% CI)	P-value
**Age group (in years)**			
< 30	1	1	
30–49	0.983 (0.860, 1.124)	1.103 (0.958, 1.270)	0.171
≥ 50	0.741 (0.642, .855)	0.992 (0.832, 1.182)	0.929
**SPO2**			
≥ 93	1	1	
< 93	0.648 (0.554, 0.757)	0.952 (0.779, 1.163)	0.630
**Temperature**			
≤ 37.5°C	1	1	
>37.5°C	1.087 (0.971, 1.218)	**1.135 (1.011, 1.274)**	**0.032**
**COVID-19 Severity**			
Mild	1	1	
Moderate	0.800 (0.699, 0.915)	0.966 (0.795, 1.173)	0.725
Severe	0.516 (0.441, 0.604)	**0.660 (0.501, 0.869)**	**0.003**
**Cough**			
No	1	1	
Yes	0.655 (0.583, 0.737)	**0.759 (0.630, 0.913)**	**0.004**
**Chest pain**			
No	1	1	
Yes	0.760 (0.639, 0.905)	0.990 (0.810, 1.209)	0.919
**Arthralgia**			
No	1	1	
Yes	0.892 (0.743, 1.072)	1.231 (0.981, 1.544)	0.072
**Fatigue**			
No	1	1	
Yes	0.727 (0.627, 0.842)	0.901 (0.741, 1.095)	0.294
**Headache**			
No	1	1	
Yes	0.833 (0.705, 0.983)	0.987 (0.827, 1.179)	0.887
**SOB**			
No	1		
Yes	0.645 (0.555, 0.748)	0.985 (0.799, 1.213)	0.884
**Hypertension**			
No	1	1	
Yes	0.874 (0.757, 1.011)	1.033 (0.873, 1.222)	0.703
**Diabetes Mellitus**			
No	1	1	
Yes	0.810 (0.682, 0.962)	0.950 (0.782, 1.152)	0.600

**Note:** CHR, Crude Hazard ratio; AHR, Adjusted Hazard ratio; CI, Confidence interval; *statistically significant.

#### Factors associated with clinical recovery of COVID-19

Univariate analysis at 25% level of significance was conducted and age group, sex, SPO2, fever, cough, chest pain, arthralgia, fatigue, shortness of breath, headache, hypertension and diabetes mellitus were found to be significantly associated with the clinical recovery from COVID-19 disease.

On the multiple binary logistic regression, after adjusting for other covariates, SPO2, shortness of breath and diabetes mellitus were found to be significantly associated with achievement of clinical recovery at 5% level of significance.

Both the subjective complaint of shortness of breath and the objectively measured oxygen saturation level were found to be significant determinants of achievement of clinical recovery. Accordingly, after adjusting for other covariates, the odds of achieving clinical recovery among patients with SPO2 of lower than 93% and a subjective complaint of shortness of breath were 69.8% and 64.6% lower than patients with SPO2 of 93% and above and with no shortness of breath, respectively (AOR = 0.302, 95% CI = 0.193, 0.474, p-value = 0.0001 for SPO2 and AOR = 0.354, 95% CI = 0.213, 0.590, p-value = 0.0001 for shortness of breath).

Having diabetes mellitus was associated with a 45.1% lower odds of achieving clinical recovery compared to those with no such comorbid illness (AOR = 0.549, 95% CI = 0.337, 0.894, p-value = 0.016). (**Table 3**)

**Table 3:** Results for the final multiple binary logistic regression model among patients with COVID-19 (n = 1345).

Variables	Outcome		COR(95% CI)	AOR(95% CI)	p-value
	Recovered	Not Recovered			
**Age group (in years)**					
< 30	444 (91.9)	39 (8.1)			
30–49	421 (92.1)	36 (7.9)	1.027 (0.641, 1.647)	1.622 (0.958, 2.749)	0.072
≥ 50	337 (83.2)	68 (16.8)	0.435 (0.286, 0.661)	1.254 (0.711, 2.213)	0.435
**Sex**					
Female	491 (88.2)	66 (11.8)			
Male	711 (90.2)	77 (9.8)	1.241 (0.876, 1.758)	1.445 (0.981, 2.129)	0.063
**SPO2**					
No	1008 (93.2)	73 (6.8)			
Yes	194 (73.5)	70 (26.5)	0.201 (0.140, 0.288)	**0.302 (0.193, 0.474)**	**0.0001**
**Fever**					
No	992 (90.0)	110 (10.0)			
Yes	210 (86.4)	33 (13.6)	0.706 (0.465, 1.070)	1.385 (0.822, 2.334)	0.220
**Cough**					
No	707 (93.1)	52 (6.9)			
Yes	495 (84.5)	91 (15.5)	0.400 (0.279, 0.573)	0.720 (0.449, 1.156)	0.174
**Chest pain**					
No	1058 (89.9)	119 (10.1)			
Yes	144 (85.7)	24 (14.3)	0.675 (0.421, 1.082)	1.325 (0.756, 2.322)	0.325
**Arthralgia**					
No	1074 (89.6)	125 (10.4)			
Yes	128 (87.7)	18 (12.3)	0.828 (0.489, 1.402)	1.508 (0.801, 2.839)	0.204
**Fatigue**					
No	984 (91.0)	97 (9.0)			
Yes	218 (82.6)	46 (17.4)	0.467 (0.319, 0.683)	1.034 (0.617, 1.732)	0.900
**SOB**					
No	986 (93.0)	74 (7.0)			
Yes	216 (75.8)	69 (24.2)	0.235 (0.164, 0.337)	**0.354 (0.213, 0.590)**	**0.0001**
**Headache**					
No	1041 (90.1)	115 (9.9)			
Yes	161 (85.2)	28 (14.8)	0.635 (0.407, 0.991)	0.830 (0.496, 1.388)	0.477
**Hypertension**					
No	973 (90.3)	105 (9.7)			
Yes	229 (85.8)	38 (14.2)	0.650 (0.437, 0.968)	1.090 (0.664, 1.729)	0.733
**Diabetes mellitus**					
No	1054 (90.8)	107 (9.2)			
Yes	148 (80.4)	36 (19.6)	2.396 (1.582, 3.628)	**0.549 (0.337, 0.894)**	**0.016**

N.B. Other co-morbid illnesses mentioned on the descriptive section were not further considered in bot regression model due to the small summed up frequency of these variables.

## Discussion

In this study, we assessed the characteristics and outcome profile of 1345 RT-PCR confirmed Patients with COVID-19 admitted to Millennium COVID-19 Care Center in Ethiopia from July to September, 2020. There is scarcity of research in African Patients with COVID-19 that describes the characteristics and outcome of patients and what affects it. Understanding these factors will guide policy makers in making evidence based decision. Among the study population, 71 (5.3%, 95% CI = 4.1%-6.5%) died, 72 (5.4%) were transferred and the rest 1202 (89.4%) were discharged improved. The median time to clinical recovery was 14 days. The mortality rate in our study was higher than a study conducted in Northern Ethiopia which reported in-hospital mortality rate of 0.8%. This difference can be attributed to the difference in the characteristics of the study participants. In our study all participants were symptomatic with almost a quarter (23.0%) presented with severe disease. On the other hand, in the Northern Ethiopia study three-quarter of the study participants were asymptomatic and only 114 (4.4%) presented with severe COVID-19 [2]. On the contrary, the death rate is low compared to studies from other African countries conducted in a comparable study population including a study from Democratic republic of Congo and Nigeria, where a death rate of 13.2% and 9.2% were reported, respectively [4, 5]. Similarly, considering the difference in population characteristics, the death rate in our study is relatively low as compared to another study in Nigeria which reported a mortality rate of 4.3% where the study participants were included 58.3% asymptomatic, 4.9% severe and 1.9% critical cases [3]. Studies conducted in Italy and New York also reported a death rate of up to 23% [15, 23]. But this discrepancy could be because of the low proportion of severe cases (23.0%) among our study population as compared with other countries admission pattern which favors admission of severe and critical COVID-19 cases.

On the log- rank test, less favorable recovery experience was observed among patients who were 50 years and older, those with hypertension, cardiac illness, diabetes mellitus, oxygen saturation of less than 93%, those with severe disease and those who are symptomatic particularly those who presented with fever, cough, chest pain, fatigue, shortness of breath, headache and nausea/ vomiting.

On the multivariable Cox proportional hazard model; temperature, COVID-19 severity and cough were found to be significant determinants of time to clinical recovery.

Accordingly, after adjusting for other covariates, the rate of achieving clinical recovery among patients with body temperature of above 37.5°C was 1.135 times than patients with temperature of 37.5°C and lower. This shows that those patients with an objectively recorded hyperthermia have a favorable recovery experience. In another case control study conducted in our center as well subjective report of fever by patients was found to be associated with a favorable outcome of being discharged alive. On the other hand, another study in the Center shows that having fever is associated with development of a more severe disease. This could be because having fever is an implication of competent body defense system that can lead to better outcome from the disease even though it could be associated with development of a more severe disease category [27, 28].

COVID-19 disease severity at admission was also found to be a significant determinant of time to clinical recovery. The rate of achieving clinical recovery among patients with severe COVID-19 disease at presentation was 34% lower than patients who presented with mild disease. On the other hand, there was no significant difference between moderate and mild cases in the time to clinical recovery. Severe patients are those patients with symptoms and signs that could lead to intensive care admission and that were also found to be significant predictors of unfavorable disease outcome in this study as well.

Presenting with a complaint of cough at admission was associated with a 24.1% lower rate of achieving clinical recovery compared to those patients with no such complaint on admission. This could be because those patients with cough were in a more severe disease category, which was also found to be a significant determinant of time to clinical recovery. Another study conducted in the Center on determinants of time to convalescence also showed that having symptomatic disease in general is associated with a delayed biochemical recovery [29].

On the binary logistic regression at 5% level of significance, the following factors were found to be significantly associated with disease outcome; SPO2, shortness of breath, and diabetes mellitus.

Both the subjective complaint of shortness of breath and the objectively measured oxygen saturation level were found to be significant determinants of disease outcome. Accordingly, after adjusting for other covariates, the odds of achieving clinical recovery among patients with SPO2 of lower than 93% and a subjective complaint of shortness of breath were 69.8% and 64.6% lower than patients with SPO2 of 93% and above and with no shortness of breath, respectively. This could be because shortness of breath and decreased SPO2 are manifestations of diseased lung with diminished capacity, which can be explained also by the high affinity of the SARS-COV-2 virus to attack the lung. Shortness of breath is also found to be associated with increased odds of death and also prolonged oxygen requirement among severe Patients with COVID-19 in other studies conducted in our Center [3, 4, 28, 30]. Having diabetes mellitus was associated with a 45.1% lower odds of achieving clinical recovery compared to those with no such comorbid illness. The effect of concomitant comorbid illness on disease progression is also reported to be the same in other studies [2, 31–34]. Patients with diabetes mellitus were also found to increased likelihood of having death outcome, developing symptomatic disease and more severe disease category in studies conducted in our center [27, 28, 35].

On the other hand, age which was found to be a significant determinant in other studies didn’t show any significant difference in this study [2, 4, 5, 23].

The study findings should be interpreted with the following strengths and limitations in mind. Its strength is that in addition to being one of the few studies conducted in the country, it is a significant addition to the existing literature as it reports a finding from the biggest COVID-19 Center in Ethiopia whose result could represent hospitalized patients characteristics and outcome in the country. However, it has the following limitations. Asymptomatic patients were not included and this might have resulted in an incomplete picture of the disease pattern and also an over estimation of the overall mortality rate from the disease in the country. In addition, the primary outcome (time to clinical recovery) was assessed as the time from admission to the institution up to achieving clinical recovery. Even if the beginning time might not exactly reflect the symptom onset for each patient as symptom could start at different time from admission time, we used the admission time as the beginning point as symptom onset time for most patients was not recorded due to different reasons and having a consistent measurement is mandatory to measure the outcome. The outcome measurement we used also reflects the time need to reach recovery while under medical supervision. The other limitation is, other relevant variables including body mass index, other behavioral factors and laboratory and radiologic parameters were not included in the study because these variables were not consistently collected from the participants.

## Conclusions

The average duration of time to clinical recovery was 14 days. The mortality rate of the studied population was 5.3%, this is lower than reports from other countries including Africa.

Having severe COVID-19 disease severity and presenting with cough were found to be associated with delayed clinical recovery from the disease. On the other hand, being hyperthermic is associated with shorter disease duration (faster time to clinical recovery). In addition, lower oxygen saturation and subjective complaint of shortness of breath and being diabetic were associated with unfavorable disease outcome.

Therefore, cautious management of these patients is mandatory for a better management outcome.

## Supporting information

S1 Questionnaire (ZIP)

S1 Dataset (SAV)
