# Supplementary material for: Characteristics and outcome profile of hospitalized African patients with COVID-19: The Ethiopian context
Source: PLoS One. 2021 Nov 9;16(11):e0259454. doi: 10.1371/journal.pone.0259454 (PMC8577729; doi:10.1371/journal.pone.0259454)
Supplement: S1 Questionnaire — (ZIP) [file pone.0259454.s001.zip › Questionnaire_Annex/0 Triage.docx]

**MILLENNIUM COVID CARE CENTER TRIAGE SHEET**

1. Patient information EPHI ID Number: ___________ MRN: ___________

MRN from referring institution: ___________

Name - ____________________________ Age: ______ Date & Time of arrival: _________________

Sex: ______________ Date & Time of Triage: ________________________

Address: Addis Ababa -> K/K: _____________ Kebele: __________ House no: ________

Outside AA -> Region: ___________ Zone/City: _________

Mobile No: ___________________ Family Member: _________

Mode of arrival -> □ Ambulance

1. Vital Signs

HR: BP: RR: Temp: SpO2: RBS:

1. Differentiating Factors

| Presentation | Respiratory distress  and/or  SpO_2_≤65 | Active seizure  SBP ≤ 90mmHg or absent radial pulses or  Capillary refill >2sec  SpO_2_ ≤ 93% on room air  Change in mentation | SOB  Cough  Acute chest pain  Diabetic + RBS >350mg/dL  Coughing up blood  SBP ≥ 180 or DBP ≥ 110 | Actively vomiting  Acute abdominal pain  Stable comorbidities such as Asthma/COPD, Cardiac illness, DM, hypertension  History of cancer diagnosis  Pregnant (1^st^ & 2^nd^ trimester)  Age ≥ 65 years  Previously bed ridden patient | All Other patients |
| --- | --- | --- | --- | --- | --- |
|  | Critical Case | Severe Case | Moderate | Mild with Risk | Mild case |
| Triage Destination |  |  |  |  |  |

1. If allergy to food or medicine (If so, please specify): ______________________________________
2. Treatment and investigation on triage: _______________________________________________________

N.B: for any patient triaged to critical care, communicate critical care specialist or senior resident on shift

*Triage Officer: ___________________________________________ Signature:___________________*
