# Supplementary material for: Characteristics and outcome profile of hospitalized African patients with COVID-19: The Ethiopian context
Source: PLoS One. 2021 Nov 9;16(11):e0259454. doi: 10.1371/journal.pone.0259454 (PMC8577729; doi:10.1371/journal.pone.0259454)

| **Millennium COVID-19 Care Center Ward Admission Note** | | | | | | | | | | | | | | | | | | | | | | | | | | | | | | | | | | | | | | | | | | | | | | | | | |
| --- | --- | --- | --- | --- | --- | --- | --- | --- | --- | --- | --- | --- | --- | --- | --- | --- | --- | --- | --- | --- | --- | --- | --- | --- | --- | --- | --- | --- | --- | --- | --- | --- | --- | --- | --- | --- | --- | --- | --- | --- | --- | --- | --- | --- | --- | --- | --- | --- | --- |
| **COVID-19 severity score**: Mild Moderate Severe | | | | | | | | | | | | | | | | | | | | | | | | | | | | | | | | | | | | | | | | | | | | | | | | | |
| **Name:** | | | | | | | | | | | | | | | | | | | | | | | | | | | | | | | **MRN :** | | | | | | | | | | | | | | | | | | |
| **Date of Admission:** | | | | | | | | | | | | | | | |  | | | | | | | | | | | | | | |  | | | | | | | | | | | | | | | | | | |
| **DEMOGRAPHICS** | | | | | | | | | | | | | | | | | | | | | | | | | | | | | | | | | | | | | | | | | | | | | | | | | |
| **Sex :** | - Male | | | | | | | - Female | | | | | | | | | | | | **Age :** _________ years | | | | | | | | | | | | | | | | | | | | | | | | | | | | | |
| **Health care worker ?** | | | | | | - Yes | | | | - No | | | | | | | - Unknown | | | | | | | **Laboratory Worker?** | | | | | | | | | | | | | | | | | | - Yes | | | | | | - No | |
| **Pregnant ?** | | - Yes | | | | | - No | | | | | - Unknown | | | | | | | | | - N/A | | | | | **If yes: Gestational weeks assessment** **[___][___]** weeks | | | | | | | | | | | | | | | | | | | | | | | |
| **How patient contracted disease** | | | | □ Contact with a dxed person | | | | | | | | | □ Works in a center caring for COVID-19 pts. | | | | | | | | | | | | | | □ Hx. of travel outside Ethiopia | | | | | | | | | | □ Contact with traveler | | | | | | □ Information Currently Unavailable | | | | | | |
| **DATE OF ONSET AND ADMISSION VITAL SIGNS (first available data at presentation/admission)** | | | | | | | | | | | | | | | | | | | | | | | | | | | | | | | | | | | | | | | | | | | | | | | | | |
| **Symptom onset** (date of first/earliest symptom) [_D_][_D_]/[_M_][_M_]/[_2_][_0_][_Y_][_Y_] | | | | | | | | | | | | | | | | | | | | | | | | | | | | | | | | | | | | | | | | | | | | | | | | | |
| **Temperature** [___][___]**.**[___]°C **Heart rate** [___][___][___]beats/min **Respiratory rate** [___][___]breaths/min | | | | | | | | | | | | | | | | | | | | | | | | | | | | | | | | | | | | | | | | | | | | | | | | | |
| **BP** [___] [___] [___](systolic) [___][___][___](diastolic) mmHg | | | | | | | | | | | | | | | | | | | | | | | | | | | | | **Severe dehydration ?** | | | | | | | | | | - Yes | | - No | | | | | | - Unknown | | |
| **Capillary refill time > 2 seconds** | | | | | | | | | | | | | | | | | | - Yes | | | | | - No | | | | | - Unk | | | | **GCS ______/15 E V M** | | | | | | | | | | | | | | | | | |
| **Oxygen saturation:** [__][__][__]% on | | | | | | | | | | | | | | - room air | | | | | | | | | - oxygen therapy | | | | | | | | | | | | | - unknown | | | | | | | | | | | | | |
| **Nutritional Status** | | | | | Height: | | | | | | | | | | | | | | Weight: | | | | | | | | | | | | | | BMI: | | | | | | | | | | | MUAC: | | | | | |
|  | | | | | | | | | | | | | | | | | | | | | | | | | | | | | | | | | | | | | | | | | | | | | | | | | |
| **CO-MORBIDITIES (existing prior to admission) (Unk = Unknown)** | | | | | | | | | | | | | | | | | | | | | | | | | | | | | | | | | | | | | | | | | | | | | | | | | |
| Chronic cardiac disease (not hypertension) | | | | | | | | | | | - Yes | | | | - No | | | | | | - Unk | | | | Current Smoking | | | | | | | | | | | | | | | - Yes | | | | | | - No | | | - Unk |
| Hypertension | | | | | | | | | | | - Yes | | | | - No | | | | | | - Unk | | | | Shisha Smoking | | | | | | | | | | | | | | | - Yes | | | | | | - No | | | - Unk |
| Diabetes | | | | | | | | | | | - Yes | | | | - No | | | | | | - Unk | | | | Khat Chewing | | | | | | | | | | | | | | | - Yes | | | | | | - No | | | - Unk |
| Chronic pulmonary disease | | | | | | | | | | | - Yes | | | | - No | | | | | | - Unk | | | | Tuberculosis | | | | | | | | | | | | | | | - Yes | | | | | | - No | | | - Unk |
| Asthma | | | | | | | | | | | - Yes | | | | - No | | | | | | - Unk | | | | Asplenia | | | | | | | | | | | | | | | - Yes | | | | | | - No | | | - Unk |
| Chronic kidney disease | | | | | | | | | | | - Yes | | | | - No | | | | | | - Unk | | | | Malignant Neoplasm | | | | | | | | | | | | | | | - Yes | | | | | | - No | | | - Unk |
| Chronic liver disease | | | | | | | | | | | - Yes | | | | - No | | | | | | - Unk | | | | Other | | | | | | | | | | | | | | | - Yes | | | | | | - No | | | - Unk |
| Chronic neurological disorder | | | | | | | | | | | - Yes | | | | - No | | | | | | - Unk | | | | If yes, specify _____________________________________ | | | | | | | | | | | | | | | | | | | | | | | | |
| HIV | | | Yes- on ART | | | | | | Yes-not on ART | | | | | | | | | | | | | Yes-viral load: | | | | | | | | | | | | | Yes-CD4 count: | | | | | | | | | | No | | | | Unk |
| **PRE-ADMISSION & CHRONIC MEDICATION Were any of the following taken within 14 days of admission** | | | | | | | | | | | | | | | | | | | | | | | | | | | | | | | | | | | | | | | | | | | | | | | | | |
| **Angiotensin converting enzyme inhibitors (ACE Inhibitors)** | | | | | | | | | | | | | | | | | | | | | | | | | | | | | | - Yes | | | | - No | | | | - Unknown | | | | | | | | | | | |
| **Angiotensin II receptor blockers** (**ARBs)** | | | | | | | | | | | | | | | | | | | | | | | | | | | | | | - Yes | | | | - No | | | | - Unknown | | | | | | | | | | | |
| **Non-steroidal anti-inflammatory (NSAID)?** | | | | | | | | | | | | | | | | | | | | | | | | | | | | | | - Yes | | | | - No | | | | - Unknown | | | | | | | | | | | |
| **Other Drugs (specify)** | | | | | | | | | | | | | | | | | | | | | | | | | | | | | | | | | | | | | | | | | | | | | | | | | |
| 1. | | | | | | | | | | | | | | | | | | | | |  | | | | | | | | | | | | | | | | | | | | | | | | | | | | |
| **2.** | | | | | | | | | | | | | | | | | | | | |  | | | | | | | | | | | | | | | | | | | | | | | | | | | | |
| **3.** | | | | | | | | | | | | | | | | | | | | |  | | | | | | | | | | | | | | | | | | | | | | | | | | | | |
| **4.** | | | | | | | | | | | | | | | | | | | | |  | | | | | | | | | | | | | | | | | | | | | | | | | | | | |


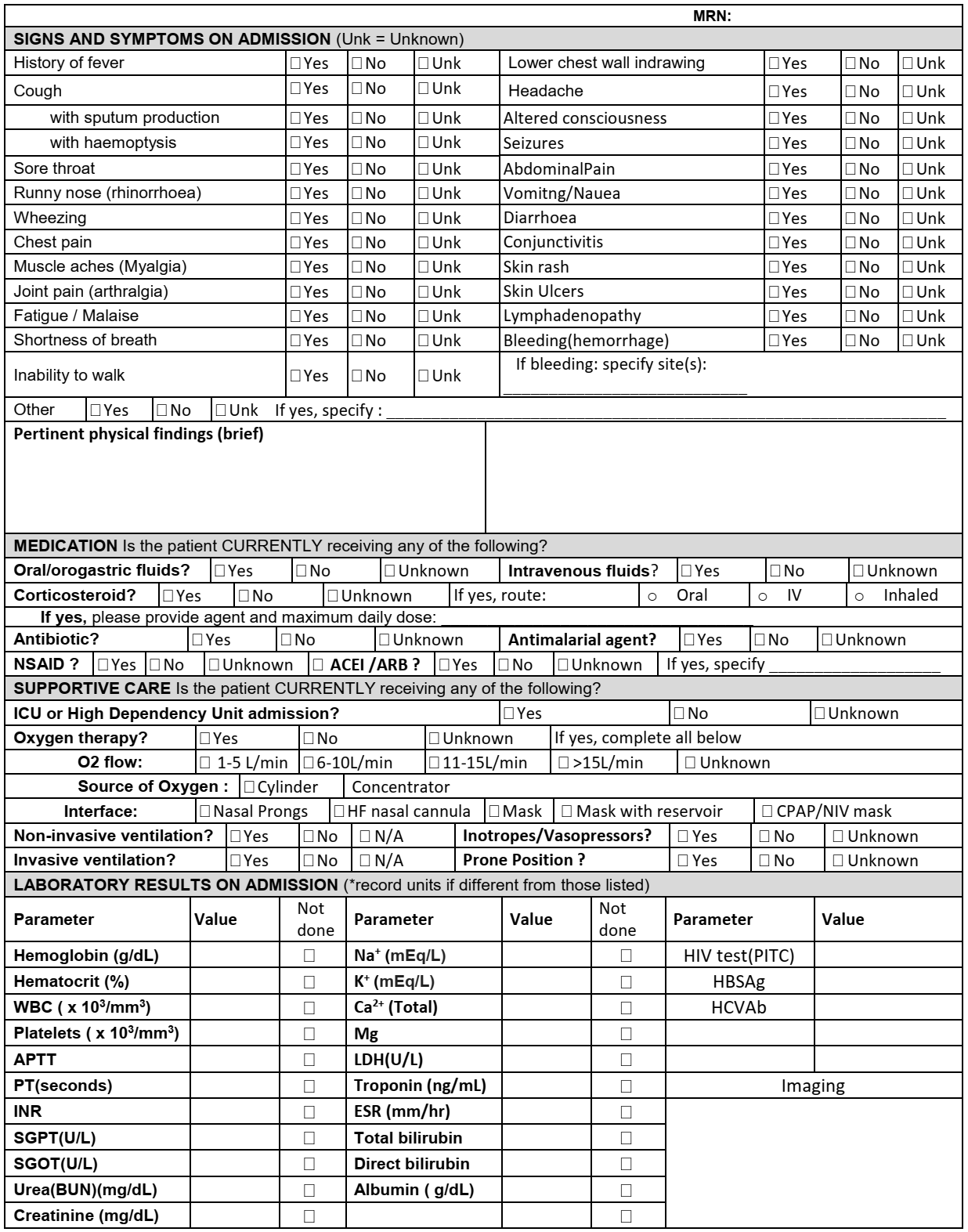

Supplement: S1 Questionnaire — (ZIP) [file pone.0259454.s001.zip › Questionnaire_Annex/1 Ward Admission.docx]
