# Supplementary material for: Characteristics and outcome profile of hospitalized African patients with COVID-19: The Ethiopian context
Source: PLoS One. 2021 Nov 9;16(11):e0259454. doi: 10.1371/journal.pone.0259454 (PMC8577729; doi:10.1371/journal.pone.0259454)
Supplement: S1 Questionnaire — (ZIP) [file pone.0259454.s001.zip › Questionnaire_Annex/4 ICU Admission.docx]

| **MCCC – ICU ADMISSION NOTE WHO COVID-19 severity score**: **Mild Moderate Severe** | | | | | | | | | | | | | | | | | | | | | | | | | | | | | | | | | | | | | | | | | | | | | | | | | | | | | | | | | | | | | | | | | | | | | | | | |
| --- | --- | --- | --- | --- | --- | --- | --- | --- | --- | --- | --- | --- | --- | --- | --- | --- | --- | --- | --- | --- | --- | --- | --- | --- | --- | --- | --- | --- | --- | --- | --- | --- | --- | --- | --- | --- | --- | --- | --- | --- | --- | --- | --- | --- | --- | --- | --- | --- | --- | --- | --- | --- | --- | --- | --- | --- | --- | --- | --- | --- | --- | --- | --- | --- | --- | --- | --- | --- | --- | --- | --- | --- |
| **SOCIO-DEMOGRAPHIC** | | | | | | | | | | | | | | | | | | | | | | | | | | | | | | | | | | | | | | | | | | | | | | | | | | | | | | | | | | | | | | | | | | | | | | | | |
| **Name:** | | | | | | | | | | | | | | | | | | | | | | | | | | | | | | | | | | | | | | | | | | | | | **MRN :** | | | | | | | | | | | | | | | | | | | | | | | | | | | |
| **Date of Admission:** | | | | | | | | | | | | | | | **Sex :** | | | | | | | | | | Male | | | | | | | | | | | | | | | | Female | | | | | | | | | | | | **Age :** _________ years | | | | | | | | | | | | | | | | | | | |
| **Health care worker ?** | | | | | | | - Yes | | | | | | | - No | | | | | | | | | - Unknown | | | | | | | | | | | **Laboratory Worker?** | | | | | | | | | | | | | | | | | | - Yes | | | | | | | | - No | | | | | | | | | | | | |
| **Pregnant ?** | | - Yes | | | | | | | - No | | | | | | | | | - Unknown | | | | | | | | | | | | | - N/A | | | | | **If yes: Gestational weeks assessment** **[___][___]** weeks | | | | | | | | | | | | | | | | | | | | | | | | | | | | | | | | | | | | |
| **How patient contracted disease** | | | | - Contact with dxed Person | | | | | | | | | | | | | | | - Works in a center caring for COVID-19 patients | | | | | | | | | | | | | | | | | | | | - Hx of travel outside Ethiopia | | | | | | | | | | | | - Contact with traveler | | | | | | | | | | | | | □ Currently unavailable information | | | | | | | | |
| **DATE OF ONSET AND ADMISSION VITAL SIGNS (first available data at presentation/admission)** | | | | | | | | | | | | | | | | | | | | | | | | | | | | | | | | | | | | | | | | | | | | | | | | | | | | | | | | | | | | | | | | | | | | | | | | |
| **Symptom onset** (date of first/earliest symptom) [_D_][_D_]/[_M_][_M_]/[_2_][_0_][_Y_][_Y_] | | | | | | | | | | | | | | | | | | | | | | | | | | | | | | | | | | | | | | | | | | | | | | | | | | | | | | | | | | | | | | | | | | | | | | | | |
| **Temperature** [___][___]**.**[___]°C **Heart rate** [___][___][___]beats/min **Respiratory rate** [___][___]breaths/min | | | | | | | | | | | | | | | | | | | | | | | | | | | | | | | | | | | | | | | | | | | | | | | | | | | | | | | | | | | | | | | | | | | | | | | | |
| **BP** [___] [___] [___](systolic) [___][___][___](diastolic) mmHg | | | | | | | | | | | | | | | | | | | | | | | | | | | | | | | | | | | | | | | | **Severe dehydration ?** | | | | | | | | | | | | | | | | | - Yes | | | | | | - No | | | | | - Unknown | | | | |
| **Capillary refill time > 2 seconds** | | | | | | | | | | | | - Yes | | | | | | | | | | | | - No | | | | | | | | | - Unknown | | | | | | | | | | | | | **GCS ______/15 E V M** | | | | | | | | | | | | | | | | | | | | | | | | | | |
| **Oxygen saturation:** [__][__][__]% on | | | | | | | | | | | | | | | | | | | - room air | | | | | | | | | | | - INO_2_  ( L) | | | | | | | | | | | | | | - FMO_2_ ( L) | | | | | | | | | | | | | | | | | | | | | | | | | | | | |
| **Nutritional Status** | | | | | | Height: | | | | | | | | | | | | | | | | | | | | | | | Weight: | | | | | | | | | | | | | | BMI: | | | | | | | | | | | | | MUAC: | | | | | | | | | | | | | | | | |
| **Pertinent physical findings (brief)** | | | | | | | | | | | | | | | | | | | | | | | | | | | | | | | | | | | | | | | | | | | | | | | | | | | | | | | | | | | | | | | | | | | | | | | | |
| **CO-MORBIDITIES (existing prior to admission) (Unk = Unknown)** | | | | | | | | | | | | | | | | | | | | | | | | | | | | | | | | | | | | | | | | | | | | | | | | | | | | | | | | | | | | | | | | | | | | | | | | |
| Chronic cardiac disease | | | | | | | | | | | | | | | | - Yes | | | | | | - No | | | | | | | | | - Unk | | | | Tuberculosis | | | | | | | | | | | | | | | | | | | | | | | | | | | - Yes | | | | - No | | | | | - Unk | |
| Hypertension | | | | | | | | | | | | | | | | - Yes | | | | | | - No | | | | | | | | | - Unk | | | | Asplenia | | | | | | | | | | | | | | | | | | | | | | | | | | | - Yes | | | | - No | | | | | - Unk | |
| Chronic pulmonary disease | | | | | | | | | | | | | | | | - Yes | | | | | | - No | | | | | | | | | - Unk | | | | Malignant Neoplasm | | | | | | | | | | | | | | | | | | | | | | | | | | | - Yes | | | | - No | | | | | - Unk | |
| Asthma | | | | | | | | | | | | | | | | - Yes | | | | | | - No | | | | | | | | | - Unk | | | | Current Cigarette Smoking | | | | | | | | | | | | | | | | | | | | | | | | | | | - Yes | | | | - No | | | | | - Unk | |
| Chronic kidney disease | | | | | | | | | | | | | | | | - Yes | | | | | | - No | | | | | | | | | - Unk | | | | Current Shisha Smoking | | | | | | | | | | | | | | | | | | | | | | | | | | | - Yes | | | | - No | | | | | - Unk | |
| Chronic liver disease | | | | | | | | | | | | | | | | - Yes | | | | | | - No | | | | | | | | | - Unk | | | | Khat Chewing | | | | | | | | | | | | | | | | | | | | | | | | | | | - Yes | | | | - No | | | | | - Unk | |
| Chronic neurological disorder | | | | | | | | | | | | | | | | - Yes | | | | | | - No | | | | | | | | | - Unk | | | | Other | | | | | | | | | | | | | | | | | | | | | | | | | | | - Yes | | | | - No | | | | | - Unk | |
| Diabetes | | | | | | | | | | | | | | | | - Yes | | | | | | - No | | | | | | | | | - Unk | | | | If yes, specify: | | | | | | | | | | | | | | | | | | | | | | | | | | | | | | | | | | | | | |
| HIV | - Yes- on ART | | | | | | | | | - Yes-not on ART | | | | | | | | | | | | | | | | | | Yes:CD4 count | | | | | | | | | | | | | | | | | | | Yes: Viral Load | | | | | | | | | | | | | | | | | | | | - No | | | | | - Unk |
| **PRE-ADMISSION & CHRONIC MEDICATION Were any of the following taken within 14 days of admission** | | | | | | | | | | | | | | | | | | | | | | | | | | | | | | | | | | | | | | | | | | | | | | | | | | | | | | | | | | | | | | | | | | | | | | | | |
| **Angiotensin converting enzyme inhibitors (ACE Inhibitors)** | | | | | | | | | | | | | | | | | | | | | | | | | | | | | | | | | | | | | | | | | | - Yes | | | | | | - No | | | | | | | - Unknown | | | | | | | | | | | | | | | | | |
| **Angiotensin II receptor blockers** (**ARBs)** | | | | | | | | | | | | | | | | | | | | | | | | | | | | | | | | | | | | | | | | | | - Yes | | | | | | - No | | | | | | | - Unknown | | | | | | | | | | | | | | | | | |
| **Non-steroidal anti-inflammatory (NSAID)?** | | | | | | | | | | | | | | | | | | | | | | | | | | | | | | | | | | | | | | | | | | - Yes | | | | | | - No | | | | | | | - Unknown | | | | | | | | | | | | | | | | | |
| **Other Drugs (specify)** | | | | | | | | | | | | | | | | | | | | | | | | | | | | | | | | | | | | | | | | | | | | | | | | | | | | | | | | | | | | | | | | | | | | | | | | |
| 1. | | | | | | | | | | | | | | | | | | | | | | | | | | | | | | | 4 | | | | | | | | | | | | | | | | | | | | | | | | | | | | | | | | | | | | | | | | | |
| **2.** | | | | | | | | | | | | | | | | | | | | | | | | | | | | | | | 5 | | | | | | | | | | | | | | | | | | | | | | | | | | | | | | | | | | | | | | | | | |
| **3.** | | | | | | | | | | | | | | | | | | | | | | | | | | | | | | | 6 | | | | | | | | | | | | | | | | | | | | | | | | | | | | | | | | | | | | | | | | | |
| **MEDICATION** Is the patient CURRENTLY receiving any of the following? | | | | | | | | | | | | | | | | | | | | | | | | | | | | | | | | | | | | | | | | | | | | | | | | | | | | | | | | | | | | | | | | | | | | | | | | |
| **Oral/orogastric fluids?** | | | | | | | | - Yes | | | | | | | | | - No | | | | | | | | | | - Unknown | | | | | | | | | | **Intravenous fluids**? | | | | | | | | | | | | | | | | - Yes | | | | | | | | - No | | | | | | | | | - Unknown | | |
| **Corticosteroid?** | | | - Yes | | | | | | | | - No | | | | | | | | | - Unknown | | | | | | | | | | | | If yes, route: | | | | | | | | | | | | | | | | | - Oral | | | | | | | | | - IV | | | | | | | | | | | - Inhaled | | | |
| **If yes,** please provide agent and maximum daily dose: __________________________________ | | | | | | | | | | | | | | | | | | | | | | | | | | | | | | | | | | | | | | | | | | | | | | | | | | | | | | | | | | | | | | | | | | | | | | | | |
| **Antibiotic?** | | | | | - Yes | | | | | | | | - No | | | | | | | | | | | | | - Unknown | | | | | | | | | | | | **Antimalarial agent?** | | | | | | | | | | | | | | | | - Yes | | | | | - No | | | | | | - Unknown | | | | | | | |
| **LABORATORY RESULTS ON ADMISSION** (*record units if different from those listed) | | | | | | | | | | | | | | | | | | | | | | | | | | | | | | | | | | | | | | | | | | | | | | | | | | | | | | | | | | | | | | | | | | | | | | | | |
| ECG interpretation: | | | | | | | | | | | | | | | | | | | | | CXR findings: | | | | | | | | | | | | | | | | | | | | | | | | | | | | | POCUS: | | | | | | | | | | | | | | | | | | | | | | |
