# Supplementary material for: Characteristics and outcome profile of hospitalized African patients with COVID-19: The Ethiopian context
Source: PLoS One. 2021 Nov 9;16(11):e0259454. doi: 10.1371/journal.pone.0259454 (PMC8577729; doi:10.1371/journal.pone.0259454)
Supplement: S1 Questionnaire — (ZIP) [file pone.0259454.s001.zip › Questionnaire_Annex/9 Investigation Summary.docx]

| **MRN:**  I_*_*_I I_*_*_I I_*_*_I I_*__*I I_*_*_I | | | | | | | | | | | | | | | |
| --- | --- | --- | --- | --- | --- | --- | --- | --- | --- | --- | --- | --- | --- | --- | --- |
| **Millennium COVID 19 Care Center – Patient Investigation Summary** | | | | | | | | | | | | | | | |
| **Patient Investigation Summary** | | | | | | | | | | | | | | | |
| Date of admission: | | Week/s of admission: | | | 1^st^ | | 2nd | | 3rd | 4th | | 5th | | 6th | 7th |
| Name : ____________________________________ Age :_______ Sex: _______ Ward : ________ Bed No:______ | | | | | | | | | | | | | | | |
| **Parameter** | **Date** | | | | | | | | | | | | | | |
|  |  | |  |  | |  | |  | | |  | |  | | |
| Hemoglobin (g/dL) |  | |  |  | |  | |  | | |  | |  | | |
| Hematocrit (%) |  | |  |  | |  | |  | | |  | |  | | |
| WBC ( x 10^3^/mm^3^) |  | |  |  | |  | |  | | |  | |  | | |
| Neut (%)  ANC |  | |  |  | |  | |  | | |  | |  | | |
| Lymph(%)  TLC |  | |  |  | |  | |  | | |  | |  | | |
| NLR (ANC/TLC) |  | |  |  | |  | |  | | |  | |  | | |
| Plt ( x 10^3^/mm^3^) |  | |  |  | |  | |  | | |  | |  | | |
| ESR (mm/hr) |  | |  |  | |  | |  | | |  | |  | | |
| CRP |  | |  |  | |  | |  | | |  | |  | | |
| BUN (mg/dL) |  | |  |  | |  | |  | | |  | |  | | |
| Cr (mg/dL) |  | |  |  | |  | |  | | |  | |  | | |
| ALT/SGPT(U/L) |  | |  |  | |  | |  | | |  | |  | | |
| AST/SGOT(U/L) |  | |  |  | |  | |  | | |  | |  | | |
| ALP |  | |  |  | |  | |  | | |  | |  | | |
| Total bilirubin |  | |  |  | |  | |  | | |  | |  | | |
| Direct bilirubin |  | |  |  | |  | |  | | |  | |  | | |
| Albumin ( g/dL) |  | |  |  | |  | |  | | |  | |  | | |
| APTT |  | |  |  | |  | |  | | |  | |  | | |
| PT(seconds) |  | |  |  | |  | |  | | |  | |  | | |
| INR |  | |  |  | |  | |  | | |  | |  | | |
| Na^+^ **(mEq/L)** |  | |  |  | |  | |  | | |  | |  | | |
| K^+^ **(mEq/L)** |  | |  |  | |  | |  | | |  | |  | | |
| Ca^2+^ (Total) |  | |  |  | |  | |  | | |  | |  | | |
| Mg^2+^ |  | |  |  | |  | |  | | |  | |  | | |
| LDH(U/L) |  | |  |  | |  | |  | | |  | |  | | |
| Troponin (ng/mL) |  | |  |  | |  | |  | | |  | |  | | |
|  |  | |  |  | |  | |  | | |  | |  | | |
|  |  | |  |  | |  | |  | | |  | |  | | |
|  |  | |  |  | |  | |  | | |  | |  | | |
| Imaging |  | | | | | |  | | | | | | | | |

| **MRN:**  I_*_*_I I_*_*_I I_*_*_I I_*__*I I_*_*_I | | | | | | | | | | | | | | | |
| --- | --- | --- | --- | --- | --- | --- | --- | --- | --- | --- | --- | --- | --- | --- | --- |
| **Millennium COVID 19 Care Center – Patient Investigation Summary** | | | | | | | | | | | | | | | |
| **Patient Investigation Summary** | | | | | | | | | | | | | | | |
| Date of admission: | | Week/s of admission: | | | 1^st^ | | 2nd | | 3rd | 4th | | 5th | | 6th | 7th |
| Name : ____________________________________ Age :_______ Sex: _______ Ward : ________ Bed No:______ | | | | | | | | | | | | | | | |
| **Parameter** | **Date** | | | | | | | | | | | | | | |
|  |  | |  |  | |  | |  | | |  | |  | | |
| Hemoglobin (g/dL) |  | |  |  | |  | |  | | |  | |  | | |
| Hematocrit (%) |  | |  |  | |  | |  | | |  | |  | | |
| WBC ( x 10^3^/mm^3^) |  | |  |  | |  | |  | | |  | |  | | |
| Neut (%)  ANC |  | |  |  | |  | |  | | |  | |  | | |
| Lymph(%)  TLC |  | |  |  | |  | |  | | |  | |  | | |
| NLR (ANC/TLC) |  | |  |  | |  | |  | | |  | |  | | |
| Plt ( x 10^3^/mm^3^) |  | |  |  | |  | |  | | |  | |  | | |
| ESR (mm/hr) |  | |  |  | |  | |  | | |  | |  | | |
| CRP |  | |  |  | |  | |  | | |  | |  | | |
| BUN (mg/dL) |  | |  |  | |  | |  | | |  | |  | | |
| Cr (mg/dL) |  | |  |  | |  | |  | | |  | |  | | |
| ALT/SGPT(U/L) |  | |  |  | |  | |  | | |  | |  | | |
| AST/SGOT(U/L) |  | |  |  | |  | |  | | |  | |  | | |
| ALP |  | |  |  | |  | |  | | |  | |  | | |
| Total bilirubin |  | |  |  | |  | |  | | |  | |  | | |
| Direct bilirubin |  | |  |  | |  | |  | | |  | |  | | |
| Albumin ( g/dL) |  | |  |  | |  | |  | | |  | |  | | |
| APTT |  | |  |  | |  | |  | | |  | |  | | |
| PT(seconds) |  | |  |  | |  | |  | | |  | |  | | |
| INR |  | |  |  | |  | |  | | |  | |  | | |
| Na^+^ **(mEq/L)** |  | |  |  | |  | |  | | |  | |  | | |
| K^+^ **(mEq/L)** |  | |  |  | |  | |  | | |  | |  | | |
| Ca^2+^ (Total) |  | |  |  | |  | |  | | |  | |  | | |
| Mg^2+^ |  | |  |  | |  | |  | | |  | |  | | |
| LDH(U/L) |  | |  |  | |  | |  | | |  | |  | | |
| Troponin (ng/mL) |  | |  |  | |  | |  | | |  | |  | | |
|  |  | |  |  | |  | |  | | |  | |  | | |
|  |  | |  |  | |  | |  | | |  | |  | | |
|  |  | |  |  | |  | |  | | |  | |  | | |
| Imaging |  | | | | | |  | | | | | | | | |
